# Foreign

**Published:** 1841-10-23

**Authors:** 


### FOREIGN.

### UNIVERSITY COLLEGE HOSPITAL.

*Contractions of the lower extremities cured by operation.*—M. S., aged eight years, was admitted June 7, under the care of Mr. Liston, with the lower limbs in an extremely crippled state. Her mother states that she was very weakly, and backward in her growth as an infant, and was unable to sit up and walk even at the age of three years, owing to weakness in the spine; this she ascribes to her being a seven months' child. When she was in her fourth year, during teething, she had convulsions, after which contractions of the lower limbs took place, and she has never yet regained the power of sitting upright. On admission the deformity was obvious, for the child, when attempting to stand, rested on the points of the toes with the knees and thighs so much bent, that progression was impossible. The thighs could not be separated from one another. Mr. Liston, on examing the limbs, observed, that there was extreme contraction of the muscles on their posterior aspect, as well as of the adductors of the thigh, and recommended their division under the skin.

The child was laid on the table on her face; and while the limbs were forcibly extended, Mr. Liston divided the tendons of the hamstring muscles of both sides, then the tendo Achillis of the left side; and in the groins the tense cord formed by the origin of the adductor longus. After the operation the joints admitted of pretty extensive motion. In a few days the extension was kept up by placing splints under the limbs, and keeping the thighs well separated.

July 16. Jointed splints with extending screws have been applied behind the knee-joint, as also the usual apparatus to bring the heel in contact with the ground.

August 1. Since the last report the child has considerably improved, and is now able to walk on the flat of the foot with very little assistance. Her movements are necessarily awkward, as she has never, till now, learned to walk.

*Hydatid Tumour in the Loins.*—Mrs. C., aged 50, and, apparently, much older, of spare habit and healthy, was admitted August 2, under the care of Mr. Liston. She has for many years been employed in washing and mangling; has had eight children, and several miscarriages. The youngest child is now seventeen years of age. About seven years since, she first experienced some pain and weakness in the loins, which drew her attention to a swelling in the right loin. This increased slowly, and occasioned some inconvenience in her avocation. The growth of the tumour was attended by a frequent pain of a darting character, which became more severe on any exertion. Catamenia ceased five years ago, and their cessation was followed by increase in the size and painfulness of the tumour. Within the last six weeks another swelling has appeared, situated about a hand's-breadth above the original one.

On admission, the tumour presented the appearances of a chronic abscess, forming two considerable prominences between the last rib and crest of the ilium close to the spine. Fluctuation was distinct, and indicated a communication between the two swellings. Mr. Liston advised its evacuation, and entered a bistoury, in a depending point, to a considerable depth. A quantity of turbid serous fluid escaped, which was followed by portions of torn cysts and a number of hydatids to the amount of nearly a quart. These bodies were globular, varying in size from that of a walnut to a pea; they were transparent, and contained fluid of a black colour. The cyst was nearly emptied of its contents by pressure, and the opening closed by plaster, supported by a compress and bandage.

August 4. The plasters have been loosened by suppuration, and the tumour has regained its original bulk, attended by pain and slight inflammatory fever. The cyst was evidently filled by bloody effusion. Fomentations to be applied to the part. To take aperient medicines.

5. There has been a copious discharge from the wound, consisting of pus and a great number of shreds of cysts and some complete hydatids. The odour of the discharge is very similar to that of liquor amnii. The patient appears much relieved by the discharge.

14. Suppuration has continued to be profuse, and as it had not a sufficient outlet, the opening was dilated, and gave exit to several ounces of healthy purulent mattter.

17. Wound discharges much less. Patient is quite convalescent.

—

*Scirrhus of the breast, with Hydatids.*—Sarah W——, aged 42, was admitted May 14, under the same surgeon. She is married, but has never had children; has always enjoyed excellent health. Catamenia have been always, and are still, regular. Nineteen or twenty years since, she first observed a small and hard swelling in the left breast, which has been gradually increasing up to a twelve month ago, and since then has increased more rapidly: the growth has not been attended with much pain until lately.

The tumour is now as large as a hen's egg, and is situated at the lower part of the mamma towards the axilla: it has the characteristic hardness of scirrhus in some parts, and in others feels like indurated lymphatic glands. The skin is not discoloured, nor adherent to the tumour, which is also extremely moveable on the pectoral muscle: there is no retraction of the nipple. The patient complains of darting pains in the swelling reaching to the axilla, particularly on moving the arm: the glands in the axilla are unaffected, and the patient's health is very good.

Mr. Liston advised the removal of the tumour, and the patient was ordered an aperient. On the 17th the operation was performed. The patient was seated with the arm separated from the side; Mr. Liston, with a scalpel, cut down upon the tumour in the directiou of the muscular fibre. The first incision opened a cyst, from which several small globular hydatids escaped: the skin was now reflected on each side, and the cyst dissected out nearly entire. On a section of the tumour, it was found to consist of a hydatid cyst and partly of scirrhus deposit. The wound was dressed with lint and cold water until all oozing had ceased, when the edges were brought together, and retained by suture and isinglass plaster.

20. The wound was united in part, the remainder suppurating freely. Zinc lotion to be applied, and the parts supported by compresses on each side of the wound and a bandage, with a sling to support the hand and arm.

June 5. Discharged cured.

—

*Chimney-sweepers' Cancer.*—Case 1. W. C., aged 24, was admitted July 22; the same surgeon. Is a chimney-sweeper, having been apprenticed to the trade at the age of seven years.

It was two years ago when he first perceived a wart on the left side of the scrotum; this increased slowly, until it attained the size of "the top of his thumb." It was very prominent, and accompanied by frequent lancinating pains. Five weeks ago the surface broke, and discharged a quantity of offensive matter, leaving an open ulcer. On admission, the sore, which was as large as a crown-piece, presented the usual characters of cancer of this part; the surface was smooth and florid, with the edges prominent and much everted; the discharge sanious, and rather fœtid. The base was indurated, but no where adherent to the testis, which, with the cord, was perfectly free.

The inguinal glands, however, were much indurated, and have been the seat of lancinating pains since the ulceration.

July 26. Mr. Liston proceeded to remove the affected parts, and decided on including the glands in the groins, which were most probably involved in the disease.

An incision was made round the ulcer, and the included parts dissected away down to the tunica vaginalis; the incision was then carried outwards along the flexure of the groin, so as to expose the lymphatic glands. These were also dissected out carefully, but with some difficulty, owing to the glands being matted together, and having deep connections.

After the ligature of a few small vessels, lint dipped in cold water was applied, and when all oozing had ceased, the edges of the wound were approximated by some points of suture and strips of isinglass plaster, assisted by position of the limb.

28. Union has entirely failed, owing to inflammatory swelling of the part, and effusion of bloody serosity, the edges of the wound are much separated, and there is copious serous discharge. Sutures and plasters to be removed, and warm water-dressings applied.

30. Suppuration is fairly established. Zinc lotion applied, and compresses on each side of the wound in the groin, with the spica bandage. The scrotum well supported.

August 7. The wounds have granulated quickly, the tunica vaginalis being entirely covered in by the scrotum.

17. The parts are now entirely healed. The patient to be discharged.

Case 2.—James O——, aged 34, was admitted August 5, also under Mr. Liston. Is a sweep by trade, and acknowledges that he has always neglected to wash the soot off his scrotum. He first perceived a small wart on the right side of that part near the raphé, which gradually increased; and five months ago the surface cracked, and became covered by a prominent crust, which separated, and was followed by others, until open ulceration took place. He has suffered only slight aching pain.

On admission, the sore had precisely the same appearance as that in the preceding case, only that it was rather smaller, and the inguinal glands were unaffected.

August 6. Mr. Liston removed the diseased parts in this case, by raising them in a fold of the scrotum, transfixing this and cutting to the surface on each side of the ulcer. Two other warts were afterwards removed, and lint dipped in cold water applied. No attempt was made in this case to obtain union, and the wound suppurated freely. Tepid water-dressings, and the parts well supported.

17. The wound was nearly healed. To be discharged.

—

*Fatty Tumour between the roots of the fingers.*—August 12. Mr. Liston to-day removed a fatty tumour from this situation of considerable size, and extending deeply between the bones of the phalanges of the index and middle fingers.

Mr. Liston observed that this was a very unusual site for adipose swellings, although ganglia and encysted tumours were of frequent occurrence in this situation.—*London Lancet.*

---

*Empyema cured by Paracentesis.* By Bell Fletcher, M.D.—March 16, 1841. Alexander Lowe, ætat. 20, of spare habit, but up to his present illness in the enjoyment of good health, as the only attack he gives any account of is one of the measles, which he had about five years since, states that about six weeks since he was seized with violent pain in the chest, particularly in the left side, cough, difficulty of breathing, rigors, thirst, loss of appetite, headach; in fact, with all the symptoms of acute pleuritis, which he attributed to cold. He did not consult any medical gentleman, but merely took simple aperients, as salts, and such like medicines.

*Present State.*—He is now much emaciated, highly nervous, and has considerable irritative fever, with a pulse of 120 per minute, but very compressible; his countenance is expressive of the greatest anxiety and distress, and he complains of severe shooting pains in the left side of the chest; great debility; dry cough; shortness of breath, which is quick, and becomes very difficult on the least exertion, at times almost threatening suffocation. He is unable to lie on the right sight, and is obliged to be supported when in bed by several pillows.

*Examination of the Chest.*—The left side measures one inch and a half more than the right; the left nipple is half an inch higher than the right. The left side appears enlarged, and the respiratory motions are scarcely perceptible in it, whilst on the right side they appear greater than usual; the heart beats two inches to the right side of the sternum.

On percussion, a dull sound is heard all over the left side, and a very sonorous one on the right side, except anteriorly to the right side of the sternum where the heart beats.

No respiration is heard on auscultation in any part of the left side of the chest, but puerile on the right.

The same state existed without any diminution of the urgency of the symptoms, except that the pains complained of were somewhat subdued, although an active treatment, consisting of mercurials, to the extent of affecting the system, purgatives, diuretics, and blistering the left side of the chest, was persevered in up to the 1st of April, when the operation of paracentesis thoracis was performed by my friend Mr. Wilkes, as the only means of saving the patient from asphyxia. The skin was drawn forcibly upwards so as to form a valvular opening, and an incision made between the sixth and seventh ribs down to near the pleura, in the situation of a line drawn downwards from the middle of the axilla; a flattened oval trocar and canula was then passed into the side, gliding on the upper edge of the seventh rib, the trocar withdrawn, and a compressed India rubber bottle, armed with two stop-cocks, immediately fixed into the canula, so as to prevent, as much as possible, the introduction of air into the cavity of the chest. By frequent applications of the bottle in a compressed state, by means of its elasticity, somewhat more than two quarts of a clear serous fluid, of a yellow colour, which coagulated strongly on cooling, was drawn from the chest, especial care being taken during the operation to manage the stop-cocks that no air might enter the chest when removing or reapplying the bottle. On withdrawing the canula, the skin immediately resuming its natural position, closed the opening. The wound was drawn together by adhesive plaster, and a compress and light bandage applied.

During the operation, the falling of the level of the fluid was distinctly traced by the dull sound on percussion being replaced by a sonorous sound, as it receded from the upper parts of the chest, till at length the whole dulness of the side affected was substituted by a clear, tympanitic sound at the lower portion, and by one somewhat less clear, corresponding to that afforded by the opposite side on percussion at the upper portion of the chest, where, on auscultation, air was heard distinctly to enter the large tubes of the lung on inspiration; and the heart evidently approached near to its normal position, beating somewhat to the left side of the middle line of the sternum.

An anodyne was given after the operation, and the patient laid on the right side, which was borne with perfect ease, (in order that the fluid which still remained in the chest might not drain through the wound, and so prevent its uniting by first intention;) the head was raised only by a single pillow; the patient expressed great relief, and his breathing was much easier.

April 4. The case goes on well from day to day, no symptom of inflammation or other mischief presenting itself. The tympanitic sound on the left side gradually diminishes from above downwards, and is succeeded by what may be called a pulmonary sound on percussion, and the respiratory sounds are heard more extensively.

8. The patient is still progressing towards health; the normal sounds on percussion, and the respiratory sounds on auscultation, are found to increase in space. The tympanitic sound does not now reach higher than the level of the inferior edge of the sixth rib under the axilla, most of which, if not all, depends upon the position of the stomach. The left side measures one inch less than the right, and the side moves freely in the actions of respiration; and as the left lung resumes its functions, respiration is heard less intense in the right.

11. The tympanitic sound now occupies a space of only about four inches square on the outer side of the situation of the heart, which has resumed its normal position. Healthy sounds on percussion and auscultation are heard generally in other parts of the chest; the respiration, which, in the first instance, was bronchial, having taken on its natural vesicular character. The left side of the chest measures half an inch more than on the 8th. The incision is perfectly healed.

17. The patient considers himself quite well, except that occasionally he has slight pains in the anterior parts of the chest; nothing abnormal can be detected on percussion or auscultation, except that at the posterior inferior portion of the chest the sound is rather more dull, and the respiration less audible than usual; in all other parts, the state is perfectly normal.

30. The pains complained of were relieved by an anodyne embrocation; the patient is now quite well, and resumes his occupation of fluting plated articles.

May 5. The patient bears his work, which is rather hard, quite well, and is in every respect in good health, and gains flesh fast, but has had some night perspiration, for which I recommended a mixture, containing diluted sulphuric acid, and disulphate of quinine in infusion of roses.

14. Since the 5th he has worked hard, which he bears well; the night sweats are diminished, and he certainly appears in every respect quite well. The state of the chest is the same as on the 17th of April.

At the present time (Aug. 2) the patient still goes on well, and says he is in as good health as ever he was in his life.

The medicines that were given during the treatment are not of sufficient importance to mention in detail; the only thing worth mentioning respecting them, is, that mercurials were given so as to slightly affect the mouth, during the ten days following the operation.

*Ibid.*

---

*The Phallus Impudicus.* By W. C. Radley, M. D.—The phallus, as an object of superstitious worship, in the earlier ages of the world, in India and in Egypt, is matter of history; and those who have read O'Brien's learned and elaborate work on the "Round Towers of Ireland,"—those ancient Phœnicians, colossal emblems of the organ of production,—will feel a full tide of associations connected with the name of *phallus*—the Lingham, the Gaye Buhd, and Buddism of India, &c. &c. My object is to impress your readers with the fact of the phallus impudicus possessing great remedial power to allay pain in the lumbar region.

Medical botanists who expect to find medicinal powers in vegetable productions, when their sensible properties are strongly developed, will not be in the least disappointed, on approaching some old hedge side, where a phallus has become erected; for a nasal nuisance more abominable than this cannot, perhaps, be found. Carrion-stench, horrid carrion-stench, bears the closest identity to its odour, that taints the air for a considerable space around the locality of its growth; and in the one emphatic sentence of Dr. Withering, "the odour of a phallus soon pervades the whole house." Unpleasant as it is, it possesses a power of allaying pain equal to morphine.

To Mr. Hele, surgeon of Ashburton, I owe acknowledgement for the first hint of the value of this species of fungus. During a delightful ride in one of our close Devonshire lanes, Mr. Hele informed me that the Rev. Mr. Bristed had said, that "the phallus impudicus possessed diuretic powers in dropsy,"—quite enough to impel me to ascertain the fact; and although I have not found it efficacious in subduing dropsy, no substance known to me allays renal pain so well, and certain painful conditions of the loins.

*Case* 1.—November, 1830. An aged woman, confined to her bed, ill of anasarca, took various diuretic medicines for some weeks, without amendment, and applied to me. A tincture of phallus was given, in tea-spoonful doses, several times a day; the secretion of urine did not increase under its use, but the sense of weight and pain in the lumbar region was effectually removed, having distressed her for a long time before. She died at length from the effects of dropsical accumulation.

*Case* 2.—January, 1832. A Mr. Underhay was desirous of being relieved from a series of "cutting-pains" in the loins, which often prevented him from following his occupation, although a sedentary one. I gave him of the powder of phallus twenty grains, made into as many pills, with thin mucilage of acacia. He took one pill thrice a day, and was cured, voiding a considerable quantity of red gravel with the renal discharge. The theory of its action in this and some other similar cases, appears to be that the phallic remedy allays the renal spasm, and the gravel is allowed to escape with the urine, assisted by its own gravity in its descent to the ureters and bladder.

*Case* 3.—Mrs. D., ætat. 43, had ascites, complicated with œdema of the legs, and great prostration of strength. An ethereal tincture of phallus impudicus was administered without sensibly diminishing the swelling; but a painful affection of the branches of nerves springing from the lumbar region, and forming the anterior crural, was completely removed, affording the sufferer most unexpected ease in this the last trying season of her life.

*Case* 4.—Mrs. V., ætat. 40, a fine, tall, prepossessing woman, who had lived before her marriage as housekeeper in a noble family, had, to use her own words, " long been a martyr" to pain, like in its class and seat to that in the preceding cases, pervading the lumbar region, and creeping around within the superior crista of the ilium. She had twenty grains of phallus powder, in twenty pills, and was permanently relieved before the half of them was taken. No words could briefly express her surprise that ten insignificant pills should have given her more immediate and continued ease than she had enjoyed in an equal time for years, from all the varied means employed. Of what could they be made? The phallus being so repulsive I durst not tell her, but merely said it was a vegetable substance that composed them. " Well," she continued, "the medicine has been invaluable to me, and I shall recommend any one with the like pains to try it; for of all the physic which I have had the abundant means of procuring in London, and various places where I have been, nothing ever benefited me so much."

To possess a sufficient quantity of a substance of such power is desirable, but not within the reach of my means to procure. In the last twelve summers, or autumns, I have not seen a score of these phalli, and for the last two years not one has offered, so that my stock has long been exhausted: moreover, one phallus yields only from fifteen to twenty-five grains of the powder, so that one object of mine in presenting this paper is to request the favour that some one of botanical predilections will, through the penny-post, send me a supply,—not of the green phallus, because of its offensive and perishable nature, but of the dried substance; and it may be quickly dried on a heated iron plate, brick, or stone. The season for it is now, and will be rapidly passing away, the phallus being found from May to the end of September only.* In the last week I found one, after a long search, growing in the centre of a double-hedge, where it had sprung up among a covert of young oak; but in a long ride on horseback, sixty miles from Plymouth, to the vicinity of Tiverton, besides circuitous routes, not one has been found; and had there been but one on either side of the road, its powerful scent would have instantly arrested the attention of any one desiring to find it.

I may further remark, that the light-brown viscid powder, kept in a well-stopped phial, is a preparation as good as the more laboured and expensive ones. Heat soon drives off the nauseous effluvium, the smell remaining being merely herbaceous. If any of my brethren will send me a few phalli so dried, "I will content them," or at least try to do so, in any way he or they may wish.

It seems humiliating that a production of nature so offensive to our nasal sense should have the power to assuage even one pain for us; but it will occur to the naturalist how often he has gained a prize or a benison in nature under unpromising circumstances, and where he least expected one. Such a fact as a despicable phallus possessing sedative power seems to open the whole tribe of crypts to our hopeful view; that among their neglected species other medicinal agents may be found. It is not long since a medical gentleman of Dublin apprised the world, that *marcantia*, as a poultice to the abdomen, caused lymph to flow in ascites; and another of London has stated that lycopodon allays the torment of cancerous sores; these are encouraging details, and urge us to lose no time in scanning their powers, but unceasingly to persevere.—*Ibid.*

---

*Memoir of the case of a gentleman born blind, and successfully operated upon in the eighteenth year of his age; with Physiological Observations and Experiments.* By J. C. August Franz, M.D., M.R.C.S. Communicated by by Sir Benjamin C. Brodie, Bart., F.R.S.—The young gentleman who is the subject of this memoir had been affected from birth with strabismus of both eyes: the right eye was amaurotic, and the left deprived of sight by the opacity both of the crystalline lens and of its capsule. At the age of seventeen, an operation for the removal of the cataract of the left eye was performed by the author with complete success. On opening the eye for the first time, on the third day after the operation, the patient described his visual perception as being that of an extensive field of light, in which everything appeared dull, confused, and in motion, and in which no object was distinguishable. On repeating the experiment two days afterwards, he described what he saw as a number of opake watery spheres, which moved with the movements of the eye, but when the eye was at rest remained stationary, and their margins partially covered one another. Two days after this the same phenomena were ob-

* It first emerges from the ground as a white ovum. When the phallus dilates, the investing membrane is ruptured, and the phallus springs up rapidly to its full growth, and is very transient. The characters of phallus impudicus any botanical work will furnish. As a whole, it bears a close resemblance to the erect penis; paper-white colour; the body is cavernous; the glans distinct, with a depression marking the orifice of the urethra, (in humanis,) make the resemblance complete.

served, but the spheres were less opake and somewhat transparent; their movements were more steady, and they appeared to cover each other more than before. He was now for the first time capable, as he said, of looking through these spheres, and of perceiving a difference, but merely a difference, in the surrounding objects. The appearance of spheres diminished daily; they became smaller, clearer, and more pellucid, allowed objects to be seen more distinctly, and disappeared entirely after two weeks. As soon as the sensibility of the retina had so far diminished as to allow the patient to view objects deliberately without pain, ribands differently coloured were presented to his eye. These different colours he could recognise, with the exception of yellow and green, which he frequently confounded when apart, but could distinguish when both were before him at the same time. Of all colours, gray produced the most grateful sensation: red, orange, and yellow, though they excited pain, were not in themselves disagreeable; while the effect of violet and of brown was exactly the reverse, being very disagreeable, though not painful. Brown he called an ugly colour; black produced subjective colours; and white gave rise to a profusion of *muscæ volitantes.* When geometrical figures of different kinds were offered to his view, he succeeded in pointing them out correctly, although he never moved his hand directly and decidedly, but always as if feeling with the greatest caution. When a cube and a sphere were presented to him, after examining these bodies with great attention, he said that he saw a quadrangular and a circular figure, and after further consideration described the one as being a square, and the other a disc, but confessed that he had not been able to form these ideas until he perceived a sensation of what he saw in the points of his fingers, as if he really touched the objects. Subsequent experiments showed that he could not discriminate a solid body from a plane surface of similar shape; thus a pyramid placed before him, with one of its sides towards his eye, appeared as a plane triangle.

Two months after the above-mentioned operation, another was performed on both eyes, for the cure of the congenital strabismus, by the division of the tendons of the recti interni muscles, which produced a very beneficial effect on the vision of the left eye; and even the right eye, which had been amaurotic, gained some power of perceiving light, and, from being atrophied, became more prominent. Still it was only by slow degrees that the power of recognising the true forms, magnitudes, and situations of external objects was acquired. In course of time, the eye gained greater power of converging the rays of light, as was shown by the continually increasing capacity of distinct vision by the aid of spectacles of given powers.—*Proceedings of the Royal Society, from London Lancet.*

*Researches tending to prove the Non-vascularity of certain Animal Tissues, and to demonstrate the peculiar uniform mode of their Organisation and Nutrition.* By Joseph Toynbee, Esq. Communicated by Sir Benjamin C. Brodie, Bart., F.R.S.—In the introduction to this paper, the author first speaks of the process of nutrition in the animal tissues which are pervaded by ramifications of blood-vessels; pointing out the circumstance, that even in them there is a considerable extent of tissue which is nourished without being in contact with blood-vessels. The knowledge of this fact leads us to the study of the process of nutrition in the non-vascular tissues; which tissues he divides into the three following classes, namely, first, those comprehending articular cartilage, and the cartilage of the different classes of fibro-cartilage. Under the second head he comprises the cornea, the crystalline lens, and the vitreous humour; and, under the third, he arranges the epidermoid appendages, viz., the epithelium, the epidermis, nails, and claws, hoofs, hair, and bristles, feathers, horn, and teeth.

The author then proceeds to show that the due action of the organs, into the composition of which these tissues enter, is incompatible with their vascularity. In proof of the non-existence of blood-vessels in these tissues, he states that he has demonstrated, by means of injections, that the arteries, which previous anatomists had supposed to penetrate into their substance, either as serous vessels, or as red blood-vessels too minute for injection, actually terminate in veins before reaching them; he also shows that round these non-vascular tissues there are numerous vascular convolutions, large dilatations, and intricate plexuses of blood-vessels, the object of which he believes to be to arrest the progress of the blood, and to allow a larger quantity of it to circulate slowly around these tissues, so that its nutrient liquor may penetrate into and be diffused through them. The author states that all the non-vascular tissues have an analogous structure, and that they are composed of corpuscles, to which he is induced to ascribe the performance of the very important functions in the process of their nutrition, of circulating throughout, and perhaps of changing the nature of the nutrient fluid which is brought by blood-vessels to their circumference. The author then brings forward facts in proof of the active and vital properties of these corpuscles, and concludes his introduction by stating, that it appears to him that the only difference in the mode of nutrition between the vascular and the non-vascular tissues is, that, in the former, the fluid which nourishes them is derived from the blood that circulates throughout the capillaries contained in their substance; whilst, in the latter, the nutrient fluid exudes into them from the large and dlated vessels that are distributed around them; and that in both classes, the particles of which the tissues are composed derive fro n this fluid the elements which nourish them.

The author then enters on an examination of the structure and mode of nutrition of the several tissues of each of these three classes. In considering the first class, he commences with articular cartilage, which he describes at great length in the various stages of its developement, and at the different periods of life. He gives in detail the account of numerous dissections of the ovum and fœtus illustrating the first stage, during which he shows that no blood-vessels enter into the substance of any of the textures composing a joint; but that the changes its component parts undergo are effected by the nutrient fluid from the large blood-vessels, by which, at this stage, each articulation is surrounded. In the second stage of the development of articular cartilage, the author shows, by numerous dissections, the process by which the blood-vessels are extended into the substance of the epiphysal cartilage, and converge towards the attached surface of articular cartilage, and how, at the same time, blood-vessels are equally prolonged over a certain portion of its free surface. He shows that none of these blood-vessels enter the substance of the articular cartilage, and he points out that in them the arteries become continuous with the veins; first, by their terminating in a single vessel, from which the veins arise; secondly, by their forming large dilatations from which the veins originate; and, lastly, they become directly continuous with the veins in the formation of loops of various characters. In the third stage, that which is exhibited in adult life, the epiphysal cartilage is converted into osseous cancelli. These contain large blood-vessels, which are separated from the articular cartilage by a layer of bone composed of corpuscles; and the author believes that the principal source of nutrition to this tissue is the nutrient fluid which exudes into it from these vessels, by passing through the articular lamella just noticed. The free surface of adult articular cartilage is nourished by vessels which pass to a slight extent over it. The author points out the presence of fine tubes which pervade the attached portion of adult articular cartilage, to which he ascribes the functions of transmitting through its substance the nutritive fluid derived from the vessels of the cancelli. He also advances the opinion that the articular cartilage becomes thinner during the whole of life, by being gradually converted into bone.

Fibro-cartilage constitutes the second tissue of the first class. The author first enters upon an examination of its structure; and in order to arrive at some definite conclusions on this subject, whereon anatomists of all ages have so much differed, he made numerous dissections of fibro-cartilages in the different classes of animals at various periods of their development, the results of which he details. He arrives at the conclusion that this tissue is composed of cartilaginous corpuscles and of fibres; the latter preponderating in adult life, the former in infancy; and that during life the corpuscles are gradually converted into fibres. He enters at length into the question of the vascularity of these cartilages; and from a careful study of many injected specimens of man and animals at various periods of their development, the particular results of which he relates, he believes that blood-vessels are contained only in their fibrous portion, and have the function of nourishing that which is cartilaginous, and which, on account of its being subject to compression and concussion, does not contain any.

Among the second class of extra-vascular tissues, the cornea is first treated of; and its structure is described as being very lax, and as containing corpuscles only in a small quantity. The opinions in favour of its vascularity are combatted; and it is shown that the blood-vessels which converge to its attached margin, and which are the principal source of the fluid that nourishes it, are large and numerous, and that at the circumference of this tissue the arteries, without any diminution of their calibre, return in their course, and become continuous with the veins. A second set of vessels, devoted to the nutrition of the cornea, is also described; they extend to a short distance over the surface of the tissue, but do not penetrate into its substance.

The crystalline lens is described as being composed of corpuscles, of which the radiating fibres are constituted. The arteria centralis retinæ is described as ramifying over the posterior surface of the capsule, where it forms large branches; these pass round the circumference of the lens, and reach its anterior surface, at the periphery of which they become straight: the arteries terminate in loops frequently dilated, and become continuous with the veins. With respect to the vascularity of the vitreous humour, the author states that although many anatomists have, in general terms, represented the arteria centralis retinæ as giving off, in its course through this organ, minute branches into its substance, still those who have paid especial attention to the subject have not been able to find such vessels. He believes that the nutrition of this stricture is accomplished by the fluid brought to its surface by the ciliary processes of the choroid, which fluid is diffused with facility through its entire substance by means of the corpuscles of which its membrane is composed, assisted by the semifluid character of the humour.

The third class of extra-vascular tissues comprehends the epidermoid appendages. The author describes them all as composed of corpuscles, which are round and soft where they are in contact with the vascular chorion, compressed and flattened where they are farther removed from it. He points out, in the substance of the hoof of the horse, the existence of fine canals, which he supposes to conduct fluid through its mass; and he states that the perspiratory ducts of the human subject possess a structure analogous to the spiral vessels of plants. The author describes each of the tissues of this class at length, and shows that the various modifications presented by the vascular system with which each is in contact, have the sole object of enabling a large quantity of blood to approach and circulate slowly around them. He also points out, in connection with this subject, the remarkable vital properties which are possessed by these non-vascular tissues.

In concluding this paper, the author states that his object has been to establish as a law in animal physiology, that tissues are capable of being nourished, and of increasing in size, without the presence of blood-vessels within their substance. He shows the analogy which is presented between the extra-vascular animal and the extra-vascular vegetable tissues. He expresses a hope that the application to surgery of the above law, with reference to the prolongation of blood-vessels into the extra-vascular tissues during disease, and to pathology in the investigation of the nature of morbid structures, particularly of those classes which contain no blood-vessels, will not be devoid of interest, and will be productive of some advantage.—*Ibid.*

---

*On the Anatomy and Physiology of certain Structures in the Orbit, not previously described.* By J. M. FERRALL, Esq., M.R.I.A. Communicated by SIR BENJAMIN C. BRODIE, Bart., F.R.S.—The author describes a distinct fibrous tunic, which he terms the *tunica vaginalis oculi*, continuous with the tarsal cartilages and ligaments in front, and extending backwards to the bottom, or apex of the orbit; thus completely insulating the globe of the eye, and keeping it apart from the muscles which move it. The eyeball is connected with this fibrous investment by a cellular tissue, so lax and delicate as to permit an easy and gliding motion between them. The use which the author assigns to this tunic is that of protecting the eyeball from the pressure of its muscles while they are in action. This tunic is perforated at its circumference, and a few lines posterior to its anterior margin, by six openings, through which the tendons of the muscles emerge in passing to their insertions, and over which, as over pulleys, they play in their course. A consequence of this structure is, that the recti muscles become capable of giving rotatory motions to the eye without occasioning its retraction within the orbit, and without exerting injurious pressure on that organ. In those animals which are provided with a proper retractor muscle, the recti muscles are, by means of this peculiar mechanism, enabled to act as antagonists to that muscle.—*Ibid.*

---

*Case of Cancrum Oris.* By STUDENS GUYENSIS.—I was called, July 24, 1841, to Edward E———, æt. 18 months, and found the little patient feverish, hot skin, fœtid breath, tongue furred, and fœted saliva flowing profusely from the mouth; several excoriations and ulcerations of the gums and roof of the mouth, and one on the hard palate being nearly half an inch in length and two lines in breadth, and of a dark bluish tinge; the molar teeth being much decayed. I ordered him to take four grains of compound scammony powder every night and morning; and a fourth of the following mixture every four hours:—

℞. Dilute sulphuric acid, gtt. x;

Tincture of bark, ʒj;

Syrup, ʒiss.

Water sufficient for an ounce mixture. Borax and honey to be applied to the ulcerations, &c., freely.

25. The bowels had been relieved copiously three times, very offensive, the head not so hot, the ulcers much the same, had taken no nourishment, and appeared wasting. Continue the medicines, and take beef-tea, &c.

26. Certainly better; the ulcerations looking healthy and florid; bowels open twice; evacuations more natural; breath and saliva less offensive, but still profuse; aud takes sago, &c.

27. Still improving; saliva decreasing in quantity, and ulcers healing fast; bowels open. To take two grains of the compound scammony powder night and morning, and continue the mixture. He went on from this time gradually improving till July 9th, when I pronounced him well.

*Remarks.*—The blue tinge, I apprehend, might be explained by the gentleman who attended before me having given calomel, for the child had been ill for ten days before I saw it; and we see in this case the value of aperients combined with the use of tonics.

*Ibid.*

---

*Spurious Labour Pains. Effects of Uterine Irritation.*—Mrs. P., a poor woman, residing in Gray's-in-lane, the mother of three children, was seized with irregular uterine action on Wednesday, the 7th of July; in consequence of which I was called to attend. On making an examination, I found the vagina relaxed, and well lubricated with mucous; the os uteri was high in the pelvis, and rigid, being dilated to the extent of half an inch in circumference, through which, during a pain, the membranes were distinctly protruded; presentation natural. Under these circumstances I left word that I should be sent for when the labour came on. In about four hours I was called a second time: the pains had been severe and constant during my absence, yet no progress had been made. At this time a pain of more than ordinary severity came on, during which she fainted. The usual means to rouse her were assiduously applied for a quarter of an hour, without the least success. Slight convulsions began to be apparent; pulse full, and breathing rather laborious. A small basin of blood was taken from the arm: after waiting half an hour, and finding her still insensible, with the paroxysms of convulsions becoming very violent, a large quantity of blood was taken away, making from both bleedings above two pounds. Ten grains of calomel were put on the tongue, and a brisk enema thrown into the rectum. After twenty minutes she was completely roused, and had a free evacuation from the bowels. I waited an hour, and finding her continuing free from convulsions, though still complaining of severe pains, I ordered an opiate, and went home. I was disturbed at six o'clock next morning: on my arrival at the house, I learned that the uterine action had been severe during the whole night; that the convulsions had returned occasionally; that the waters had come away; and that the bowels had been moved with calomel: not the least progress had been made, although, as the women said, "she had been in strong labour all night." As she was beginning to be exhausted from want of rest, and the continued annoyance of these ineffectual pains, I gave her a second opiate, from which she enjoyed a few hours sleep. After this the pains returned for some hours, but towards night left her altogether, and in a day or two she was going about as usual, and kept pretty well, till Friday, the 20th of August, when, after a quick and easy labour, she gave birth to a fine girl, being more than a month after her two days' sufferings from spurious labour pains. This case affords a good example of the propriety of judicious delay in manual interference. During the second days' illness, Mrs. P., and a conclave of sympathising friends, urgently demanded that something should be done for her relief; but considering that there was no hæmorrhage, that the os uteri was high in the pelvis, hard, and but little dilated, and that the convulsions had nearly ceased, it was obvious that there was no symptom indicating the least risk to life: their entreaties were accordingly firmly resisted, and Mrs. P.'s mind quieted with the assurance that there was no danger, and that proper labour had not commenced, and probably would not come on for a week or two. W. C. M'E.

*Ibid.*

*Stone in the Bladder.*—A colonel of engineers, in the service of the East India Company, about thirty years ago, removed a stone from his bladder by files introduced through the urethra: the operation was very tedious, but it proves the possibility of the thing. Dr. Darwin described an apparatus which he conjectured might be used with this intention.—*Dr. Arnott's Essay.*

